# Effects of soy protein isolate and soy peptide preload on gastric emptying rate and postprandial glycemic control in healthy humans

**DOI:** 10.1186/s40101-022-00299-9

**Published:** 2022-06-27

**Authors:** Hatsumi Ueoka, Yoshiyuki Fukuba, Masako Yamaoka Endo, Toshio Kobayashi, Hironobu Hamada, Hideaki Kashima

**Affiliations:** 1 Department of Physical Analysis and Therapeutic Sciences, Graduate School of Biomedical & Health Sciences, Hiroshima University, Hiroshima, Japan; 2 School of Health Sciences, Prefectural University of Hiroshima, Hiroshima, Japan; 3 Faculty of Health and Sports Sciences, Hiroshima International University, Hiroshima, Japan; 4 School of Nursing, Graduate School of Nursing, Aichi Prefectural University, Aichi, Japan

**Keywords:** Glucose, Insulin, Soy peptide, Soy protein isolate, Gastric emptying, Oral glucose tolerance test (OGTT)

## Abstract

**Background:**

This study aims to compare the effects of soy protein isolate (SPI) and soy peptide (PEP) preload 30 min before a 75-g oral glucose tolerance test (OGTT) on the gastric emptying rate, plasma insulin, and blood glucose responses.

**Methods:**

Nine healthy young subjects were evaluated on four occasions. The participants consumed a 200-ml solution containing either 20 g of SPI or PEP in experiment 1. In experiment 2, 30 min after consuming either 20 g of SPI or PEP solutions, an OGTT was performed to evaluate the individual glycemic response. The gastric emptying rate was measured by the ^13^C-sodium acetate breath test. Blood glucose and plasma insulin were measured before and after consuming either the SPI or PEP solutions and during the OGTT.

**Results:**

In experiment 1, plasma insulin levels were higher 30 min after consuming the PEP solution than after the SPI solution. PEP resulted in a faster gastric emptying rate than SPI. In experiment 2, just before performing the OGTT, the plasma insulin response was higher for PEP than for SPI. Fifteen minutes after starting the OGTT, the blood glucose response was lower after consuming PEP than after SPI. The gastric emptying rate tended to be faster after consuming PEP than after SPI (*p* = 0.08).

**Conclusion:**

A PEP preload might be slightly more effective for the suppression of postprandial blood glucose excursion compared with SPI; thus, a PEP preload potentially induces an enhanced insulin response just before the OGTT.

## Background

Controlling postprandial blood glucose levels to remain within the normal range is important for preventing complications, such as neuropathy, renal disorders, and heart failure. The guidelines for the management of postprandial blood glucose [1] indicate that postprandial hyperglycemia is a risk factor that accelerates complications of diabetes, and recently, several dietary therapies for the prevention of postprandial hyperglycemia have been proposed [1]. The postprandial blood glucose level is determined by the interactions among insulin, glucagon, incretin hormones, and gastric emptying [2]. It is well known that postprandial hyperglycemia is induced by abolition of the initial phase insulin response (IPIR) following meal ingestion in type 2 diabetic patients [3]. Gastric emptying plays a major role in the digestion of nutrients and is an important factor for determining postprandial glycemic responses [2]. As a strategy to accelerate the IPIR immediately after meal ingestion, protein ingestion before the main meal, called protein preloading (PPL), has recently been reported [4–9].

Whey protein derived from milk protein consumption (50–55 g) 30 min prior to consuming a carbohydrate-rich meal reduced the postprandial hyperglycemic response compared with no prior whey protein consumption in patients with type 2 diabetes [6, 8]. Akhavan et al. [4] reported that even relatively small amounts of whey protein (10–40 g) were still effective to reduce the postprandial glycemic response in healthy subjects. This positive effect of whey protein preloading on the postprandial glycemic response was due to an enhanced IPIR and slower gastric emptying with increased glucose-dependent insulinotropic polypeptide (GIP), glucagon-like peptide-1 (GLP-1), and cholecystokinin (CCK) secretion preceding the main meal [4, 6, 8]. Recently, two studies reported consistent results that consumption of real food (milk) before main meals suppress the subsequent blood glucose response [10, 11]. These results are beneficial for North American and European people who frequently consume milk at particular times. By contrast, in Asian countries, soy milk has been popular since ancient times. Asians typically consume 9 to 30 g soybeans and soy products per day, which is higher than Americans [12]. In Japan, soybean is ingested on a daily basis in the form of processed foods such as tofu, miso, and natto. In addition, soy products can be safely consumed by people with lactose intolerance [13]. It is, therefore, meaningful to investigate the effect of PPL with different types of proteins.

To our knowledge, there is less evidence on other types of PPL (e.g., soy protein) than whey PPL. Two previous studies reported that PPL with soy protein (0.5 g/kg) [9] and soy protein isolate (SPI) (40 g) [7] can also reduce the postprandial blood glucose response compared with control conditions without PPL. However, to practically use PPL with soy protein, protein intake might need to be further reduced because of overall energy intake and an increase in the risk of type 2 diabetes [14, 15].

To mitigate concerns about the high-dose consumption of soy protein, soy peptide (PEP) might be a good protein source because hydrolyzed soy protein displays greater insulin secretion than intact soy protein [16]. Therefore, we hypothesized that PPL with PEP could more effectively reduce the postprandial blood glucose response compared with intact soy protein via exaggerated insulin response at the initial phase of the OGTT (i.e., IPIR). To test this hypothesis, we investigated the influence of preloading PEP and protein before the OGTT on subsequent glycemic control in healthy subjects. Moreover, to provide a further understanding of differences in the physiological response between intact soy protein and PEP, we investigated the following two factors. First, we examined whether a greater insulin response can be induced by PEP consumption than by intact soy protein consumption according to a previous study [16]. Second, we compared the gastric emptying rate between soy protein and PEP. The incretin effect is greater when the gastric emptying rate is faster [2]. These findings are expected to serve as a basis for the mechanism of postprandial glycemic control with PPL.

## Methods

### Subjects

Nine healthy young Japanese subjects (seven females and two males; age, 22 ± 3 years; height, 162 ± 5 cm; weight, 56 ± 6 kg; mean ± SD) participated in this study. The subjects were normotensive, did not smoke or take any medications, and had no history of autonomic dysfunction or cardiovascular disease. This study was approved by the Ethics Committee of the Prefectural University of Hiroshima (No. HH14004), Japan, and each subject provided written informed consent to participate prior to the commencement of the study. The subjects arrived at 08:30 in the laboratory after fasting for 10 h overnight and having abstained from strenuous exercise, alcohol, and caffeine for at least 1 day. The subjects were seated in a chair in a quiet room, where the temperature and humidity were maintained at 22 ± 1 °C and 30% ± 4%, respectively.

### Experimental design

This study consisted of two experiments, and the subjects underwent a total of four protocols. Experiment was conducted with a single-blinded, randomized, crossover design. Female subjects were scheduled for assessment during their late follicular phase because the menstrual cycle affects the gastric emptying rate and blood glucose, insulin, and GLP-1 concentrations [17, 18]. Male subjects participated a maximum of once per week.

In experiment 1, after collecting baseline blood samples and breath samples, the subjects were instructed to consume a 200-ml drink containing either 20 g of SPI powder (HD-101R, Fuji Oil Co., Ltd., Osaka, Japan) or 20 g of PEP powder (HI-NUTE AM (Fuji Oil Co., Ltd., Osaka, Japan) within 1 min; the subjects were then monitored for 120 min. In experiment 2, after collecting baseline blood samples and breath samples, the subjects were instructed to consume a 200-ml preloading drink containing either 20 g of SPI or PEP (i.e., the same drink as experiment 1). Thirty minutes later, they consumed a 225-mL drink containing 75 g of glucose (Trelan-G; Ajinomoto Pharma Co., Ltd., Tokyo, Japan) (i.e., OGTT) and subsequently rested for 120 min. Three grams of the artificial sweetener Pal Sweet (Ajinomoto Co., Ltd., Tokyo, Japan) was added to all preloading drinks for palatability. The amino acid compositions of SPI and PEP were determined using an amino acid automatic analysis method (L-8900, Hitachi High-Tech Science Co., Ltd., Tokyo, Japan) (Table 1).

**Table 1:** Amino acid composition of soy protein isolate and soy peptide (g/100 g protein)

	**Soy protein isolate**	**Soy peptide**
Alanine	3.7	3.3
Arginine	6.7	6.8
Aspartic acid	10.2	10.5
Cysteine	1.1	1.1
Glutamic acid	17.0	18.5
Glycine	3.6	3.5
Histidine ^a^	2.3	2.2
Isoleucine ^b^	4.0	3.2
Leucine ^b^	6.9	5.6
Lysine ^a^	5.5	5.5
Methionine ^a^	1.1	1.0
Phenylalanine ^a^	4.6	3.8
Proline	4.6	4.7
Serine	4.4	4.6
Threonine	3.4	3.2
Tryptophan ^a^	1.2	0.9
Tyrosine	3.4	2.9
Valine ^b^	4.2	3.4

^a^Essential amino acids

^b^Branched-chain essential amino acids

### Blood sampling

In experiment 1, capillary blood samples were collected at baseline and at 15, 30, 45, 60, 90, and 120 min after ingestion of the SPI or PEP drinks. In experiment 2, capillary blood samples were collected at baseline (30 min before the start of the OGTT and immediately before consuming the preload drink) and before (− 5 min) and during the OGTT (15, 30, 45, 60, 90, and 120 min). Capillary blood samples were collected by pricking the right index and middle fingers. Blood glucose concentrations were analyzed with a dedicated measurement device (Arkray glucocard Diameter-alpha, GT-1661; Arkray, Inc., Kyoto, Japan). Blood samples were collected into post-heparin 75-µl capillary tubes and centrifuged at 10,000 to 12,000 revolutions per min for 5 min to obtain plasma samples. The plasma samples were refrigerated at − 20 °C. Plasma insulin concentrations were measured using an enzyme immunoassay kit (Mercodia Insulin ELISA, Mercodia Co., Ltd., Uppsala, Sweden).

### Gastric emptying

The gastric emptying rate was evaluated using the ^13^C sodium acetate breath test [19]. After ^13^C-sodium acetate is administered via the oral route, it is absorbed rapidly in the small intestine (but not in the stomach), metabolized, and finally expired as [^13^CO_2_]. This breath test has been widely used as a reproducible and noninvasive measurement alternative to scintigraphy, which has the drawback of radioactive exposure [19]. In experiment 1, 100 mg of ^13^C-sodium acetate (Cambridge Isotope Laboratories, Woburn, MA, USA) was dissolved in the SPI and PEP solutions. In experiment 2, 100 mg of ^13^C-sodium acetate was dissolved in the 75-g OGTT solution. The participants were instructed to hold their breath for 10 s to allow us to obtain end-expiratory breath in a sample foil bag. Baseline breath samples were collected using a large-capacity bag (PAYLORI-BAG5 L; Fukuda Denshi, Tokyo, Japan), and during the experiment, breath samples were collected using a small-capacity bag (PAYLORI-BAG20; Fukuda Denshi). In experiment 1, breath samples were collected at baseline and at 5-, 10-, and 15-min intervals for 5–30, 30–60, and 60–120 min, respectively. In experiment 2, breath samples were collected at baseline, before (− 5 min), and during the OGTT at 5-, 10-, and 15-min intervals for 5–30, 30–60, and 60–120 min, respectively. [^13^CO_2_] enrichment in the breath was measured using an isotope ratio mass spectrometer (POCone; Otsuka Electronics, Hirakata, Japan). CO_2_ production was assumed to be 300 mmol per m^2^ body surface area per hour. Body surface area was calculated according to the weight–height formula [20]. Time courses of the percentage [^13^CO_2_] recovery per hour and the cumulative percentage of [^13^CO_2_] recovery were determined. The times when the [^13^CO_2_] recovery per hour reached a maximum (*T*_max-calc_) was calculated according to a standard analytical method [19]. This parameter is closely correlated with the gastric emptying rate obtained using a scintigraphic method [19, 21, 22] and the Wagner–Nelson method [23].

### Calculations

The peak blood glucose values and peak plasma insulin concentrations during the OGTT were evaluated. The blood glucose and plasma insulin responses were calculated as the incremental area under the curve (iAUC) above baseline values following SPI or PEP preloading.

### Data analysis

The data are expressed as the mean and standard error (SE) of the mean. The effects of time and solution on blood glucose and plasma insulin concentrations were analyzed by a two-way repeated analysis of variance. When a significant effect was detected, Dunnett’s and paired *t* post hoc tests were conducted to reveal the effects of time (the change from baseline) and solution, respectively. The effects of solution on the peak blood glucose values, peak plasma insulin concentrations, blood glucose iAUC, plasma insulin iAUC, and *T*_max-calc_ were assessed by a paired t-test. The level of statistical significance was set at *p* < 0.05. All statistical analyses were performed with SPSS PASW 18 statistics software (SPSS Inc., Chicago, IL, USA).

## Results

### Experiment 1

**Fig. 1:**
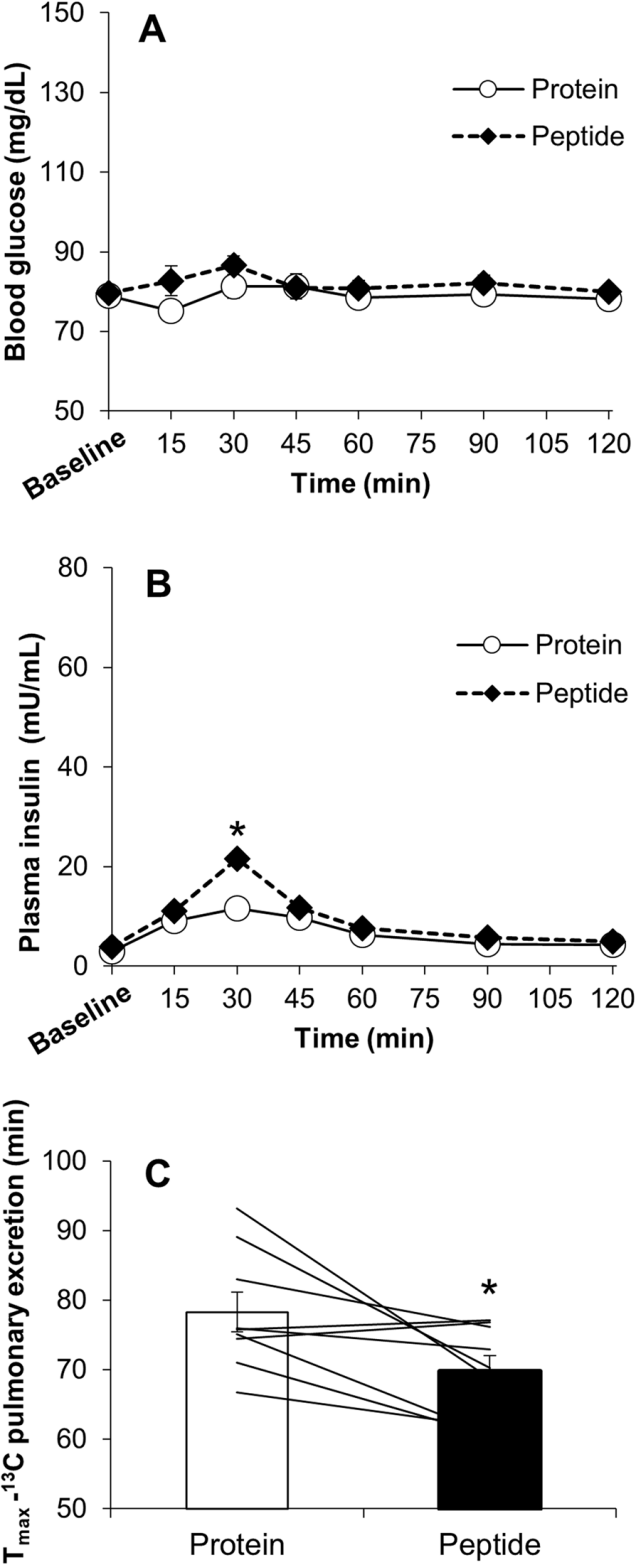
Time courses of blood glucose responses (**A**), plasma insulin responses (**B**), and the gastric emptying rate (**C**) in experiment 1. The subjects consumed a 200-ml drink containing either 20 g of soy protein isolate (protein) or its peptide (peptide) and then rested for 120 min. The lines denote individual data in C (*n* = 9). Mean ± SE. protein vs. peptide, *p < 0.05

There were no differences in blood glucose and plasma insulin concentrations between subjects consuming the two solutions at baseline. Blood glucose concentrations after the ingestion of the SPI and PEP solutions did not change from baseline values (Fig. 1A). However, the plasma insulin concentration increased significantly 30–45 min after the ingestion of SPI and PEP. Thirty minutes after consuming the soy solution, PEP resulted in higher plasma insulin concentrations than SPI (Fig. 1B).

The T_max-calc_ for PEP was shorter (i.e., faster gastric emptying rate) than that for SPI (SPI vs. PEP, 51.4 ± 2.1 vs. 43.6 ± 2.2 min, respectively, *p* < 0.05) (Fig. 1C).

### Experiment 2

**Fig. 2:**
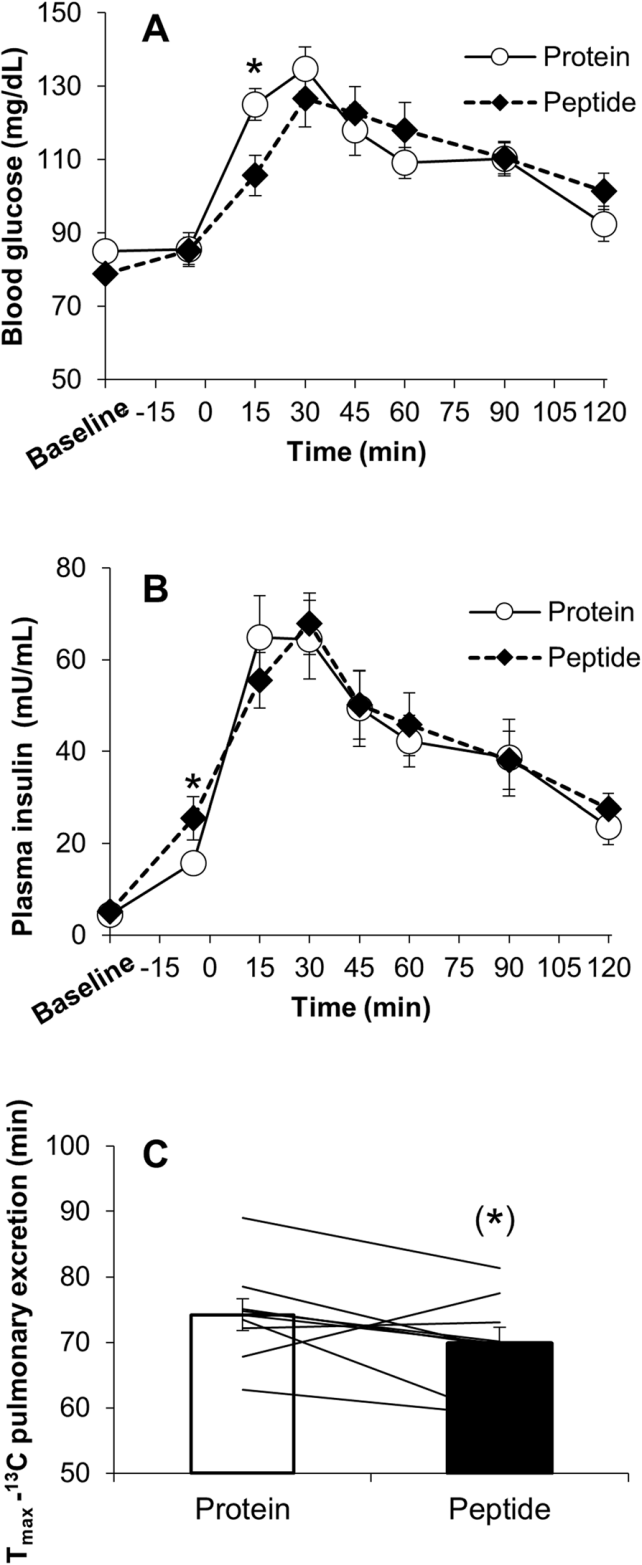
Time courses of blood glucose responses (**A**), plasma insulin responses (**B**), and the gastric emptying rate (**C**) in experiment 2. The subjects consumed a 200-ml drink containing either 20 g of soy protein isolate (protein) or its peptide (peptide) 30 min before the 75-g oral glucose tolerance test, which lasted 120 min. The lines denote individual data in C (*n* = 9). Mean ± SE. protein vs. peptide, *p < 0.05 and *p = 0.08

There were no differences in blood glucose and plasma insulin concentrations between subjects consuming the two solutions at baseline. During the 15–120 min OGTT period in both conditions, blood glucose significantly increased from baseline (Fig. 2A). Fifteen minutes after starting the OGTT, PEP resulted in a lower blood glucose response than SPI (Fig. 2A). The iAUC and peak value of blood glucose were not significantly different between the two conditions. Under both conditions, plasma insulin concentrations increased significantly from baseline at – 5 min (i.e., just before beginning the OGTT) and during the OGTT (Fig. 2B). At − 5 min, PEP resulted in higher plasma insulin concentrations than SPI (Fig. 2B). The iAUC of plasma insulin was not significantly different between the two conditions. The peak value of plasma insulin after PEP consumption tended to be greater than that after SPI consumption.

The *T*_max-calc_ for PEP tended to be shorter than that for SPI (SPI vs. PEP, 74.2 ± 2.4 vs. 69.8 ± 2.5 min, respectively, *p* = 0.08) (Fig. 2C).

## Discussion

This study was designed to explore the possibility of suppressing postprandial blood glucose by preloading with a smaller soy protein source before consuming the main meal. At the initial phase during OGTT, preloading with PEP modestly suppressed the elevation of the blood glucose response compared with SPI. This suppressive effect might lead to an exaggerated insulin response just before an OGTT via a potentially quicker gastric emptying rate and PEP-specific effects. These results suggest that when a comparable amount of a different soy protein source is consumed before eating a main meal, PEP might be slightly more effective for suppressing the postprandial glycemic response than SPI.

### Experiment 1

PEP induced a greater insulin response than SPI. This result is consistent with a previous study that compared the insulin response to the consumption of soy protein and its hydrolysates in humans [16]. Thus, PEP can potentially induce a greater insulin response than SPI, whereas its mechanism of action has not been elucidated. Nilsson et al. [24] reported that the insulin response was strongly correlated with an increase in plasma amino acid levels. In addition, when the gastric emptying rate was suppressed after strenuous resistance exercise, the elevation of plasma branched-chain amino acids was also delayed [25]. If the amount of amino acids entering the blood circulation can play a key role in accelerating the insulin response, the digestive and absorptive rate on PEP might be partially explained. Marathe et al. [2] suggest that the incretin effect is greater when the gastric emptying rate is faster and that the gastric emptying rate is responsible for approximately 35% of the incretin response in both healthy individuals and those with type 2 diabetes. In fact, PEP resulted in a quicker gastric emptying rate than SPI (Fig. 1B). Maebuchi et al. [26] reported that the degree of hydrolysis of dietary proteins can influence their absorption rate. Specifically, when soy drinks containing three different sources (protein, peptide, and amino acid mixture) with the same amino acid composition were ingested in humans, the peptide type drink showed the most rapid and significant appearances in serum amino acid concentrations among the three treatments. This finding suggests that the peptide transport system plays a certain role in the absorption of protein and peptide digestive products [27].

### Experiment 2

PEP could suppress the blood glucose response compared with SPI at the initial phase (i.e., 15 min) during the OGTT (Fig. 2A). This suppressive effect might be induced by an exaggerated insulin response just before the onset of the OGTT by PEP preloading. Our data suggest that an enhanced IPIR can lead to reduced postprandial blood glucose excursion even in healthy young participants who do not have impaired insulin secretion. Although the iAUC and peak values for blood glucose were not significantly different between preloading with SPI and PEP, PEP might modestly mitigate concerns about the high-dose consumption of preloading with soy protein.

 PEP did not show further inhibition of the glycemic response 30 min after OGTT. This result might be partly related to quicker gastric emptying in PEP than SPI following OGTT. As shown in Fig. 2C, PEP displayed a quicker (but not significantly) gastric emptying rate than SPI, which might lead to an unsustainable suppression of the blood glucose response throughout the OGTT after PEP preloading. In fact, a previous study showed that branched-chain amino acid mixture which hydrolyzed whey protein accelerates insulin secretion likewise whey protein, whereas gut hormones secretion associated with slowing gastric emptying (i.e., GLP-1) are less than whey protein [28]. Therefore, it seems to be suggested that lesser secretion of GLP-1 in PEP than SPI might be partly contributed to glycemic response 30 min following OGTT. As we did not measure gut derived hormones associated with regulation of gastric emptying, future studies should evaluate them. In addition, to further suppress the glycemic response should also focus on approaches not only to suppress initial phase of glycemic response but also to slow gastric emptying after OGTT, because gastric emptying is one of the most important factors for determining postprandial blood glucose excursion [2, 29, 30].

This study has the following limitations. First, there were only nine healthy young male and female subjects. With this small sample size, it is possible that the difference between the soy protein solutions could not be detected. In future studies, a larger sample size with sufficient statistical power should be analyzed. Second, we could not measure various gut hormones associated with the regulation of the gastric emptying rate and incretin, GLP-1 and GIP, respectively. Both of those incretin hormones have strong insulinotropic effects even though they work through different mechanisms. GIP stimulates insulin release, and GLP-1 exerts a negative feedback mechanism on gastric emptying [2]. Other hormones linked to appetite regulation, including CCK and peptide YY, also appear to influence the gastric emptying rate [2]. To further develop an understanding of different incretin responses between SPI and PEP, those gut hormones must be evaluated in future studies. Third, we could not measure soy isoflavone associated with control postprandial blood glucose. Specifically, genistein, a type of soy isoflavone, has been reported to inhibit α-glucosidase activity [31]. α-glucosidase is an enzyme that catalyzes the cleavage of glucose from disaccharides and inhibition of this enzyme has been recognized as an effective approach for lowering glucose level. In vitro, peptides extracted from soy protein have been reported to have α-glucosidase inhibitory activity [32]. Thus, suppression of α-glucosidase activity with PEP preloading may contribute to the suppression of glycemic response in the early phase of OGTT.

## Conclusion

In conclusion, PEP preloading inhibited the blood glucose response at the initial phase during the OGTT compared with SPI preloading in healthy young subjects. This slight effect might be caused by the enhanced insulin secretion prior to the onset of the OGTT. To further suppress the postprandial blood glucose response, we must focus on not only accelerating insulin secretion before eating a main meal but also subsequently slowing the gastric emptying rate.

## Data Availability

The datasets generated during the current study are not publicly available but are available from the corresponding author on reasonable request.

## Abbreviations

iAUC: Incremental area under the curve
IPIR: Initial phase insulin response
OGTT: Oral glucose tolerance test
PEP: Soy peptide
PPL: Protein preloading
SPI: Soy protein isolate
Tmax-calc: The times when the [13CO2] recovery per hour reached a maximum
